# Embryonal Rhabdomyosarcoma of the Cervix: A Rare Disease at an Uncommon Age

**DOI:** 10.7759/cureus.1864

**Published:** 2017-11-21

**Authors:** Uroosa Ibrahim, Amina Saqib, Farhan Mohammad, Juan Ding, Blerina Salman, Fady K Collado, Meekoo Dhar

**Affiliations:** 1 Department of Hematology and Oncology, Staten Island University Hospital; 2 Pulmonary/Critical Care, Staten Island University Hospital; 3 Pathology, Staten Island University Hospital; 4 Gynecology, Staten Island University Hospital; 5 Gynecologic Oncology, Staten Island University Hospital

**Keywords:** embryonal rhabdomyosarcoma, cervical cancer, vaginal bleeding, botryoid, exophytic mass

## Abstract

Embryonal rhabdomyosarcoma (RMS) is a rare type of sarcoma, primarily seen in the pediatric and adolescent population. Three subtypes of embryonal RMS are described, with the botryoid type being the most common. The incidence of this disease in adult females is 0.4% to 1% with the affected age group being patients in the third to fourth decade of life. It is exceedingly rare in patients above 40 years of age. We describe the case of a 48-year-old female, gravida 9 para 5, who presented with abnormal vaginal bleeding and an exophytic mass on examination. Given her lack of requirement of maintaining parity, she underwent radical surgery. The tumor was 8 cm in the largest dimension with a high histologic grade and some cartilaginous differentiation. Immunohistochemical stains were positive for vimentin, CD99, myogenin, and MyoD1 consistent with a diagnosis of embryonal rhabdomyosarcoma, botryoid subtype. Based on high survival rates when treated with aggressive adjuvant chemotherapy, a decision was made to treat the patient with the ARST0331 regimen. We discuss the diagnostic pathologic features of the disease, the epidemiology, and the most common presentation along with prognostic factors, treatment strategies, and outcomes.

## Introduction

Rhabdomyosarcoma (RMS) of the cervix is a rare disease entity, particularly in the adult population. The tumor is histologically subdivided into the embryonal, alveolar, sclerosing, and pleomorphic types. While embryonal is the most common histology seen, it also has three subtypes; botryoid, spindle cell, and not-otherwise-specified. Given the rarity of the disease and its histologic variants, standard treatment guidelines are not only scarce but difficult to devise [1]. Reported cases and reviews help in managing the disease based on patient characteristics and prognostic factors such as tumor bulk, the extent of spread, and lymphovascular invasion. We discuss the botryoid subtype of embryonal RMS in an older female with a bulky tumor removed in its entirety and treated with adjuvant chemotherapy. We also discuss the diagnostic pathologic features of the disease, epidemiology, presenting manifestations, prognostic factors, treatment strategies, and outcomes of therapy.

## Case presentation

A 48-year-old female was referred to the gynecology clinic with symptoms of abnormal vaginal bleeding for the prior three to four months. She was gravida 9 and para 5, with a history of one miscarriage and three elective terminations. Her last visit to a gynecologist was 13 years ago after her last pregnancy. The patient’s past history was significant for hypertension controlled with a calcium channel blocker and she had a laparoscopic ovarian cystectomy several years ago. Her family history was significant for a maternal first-degree cousin with a gynecologic malignancy (uterine or cervical) diagnosed in her 20s treated with curative surgery. On pelvic examination, she was seen to have blood clots in the vaginal canal. The cervix appeared dilated, with a protruding friable mass that was biopsied with pathology consistent with embryonal rhabdomyosarcoma.

**Figure 1:**
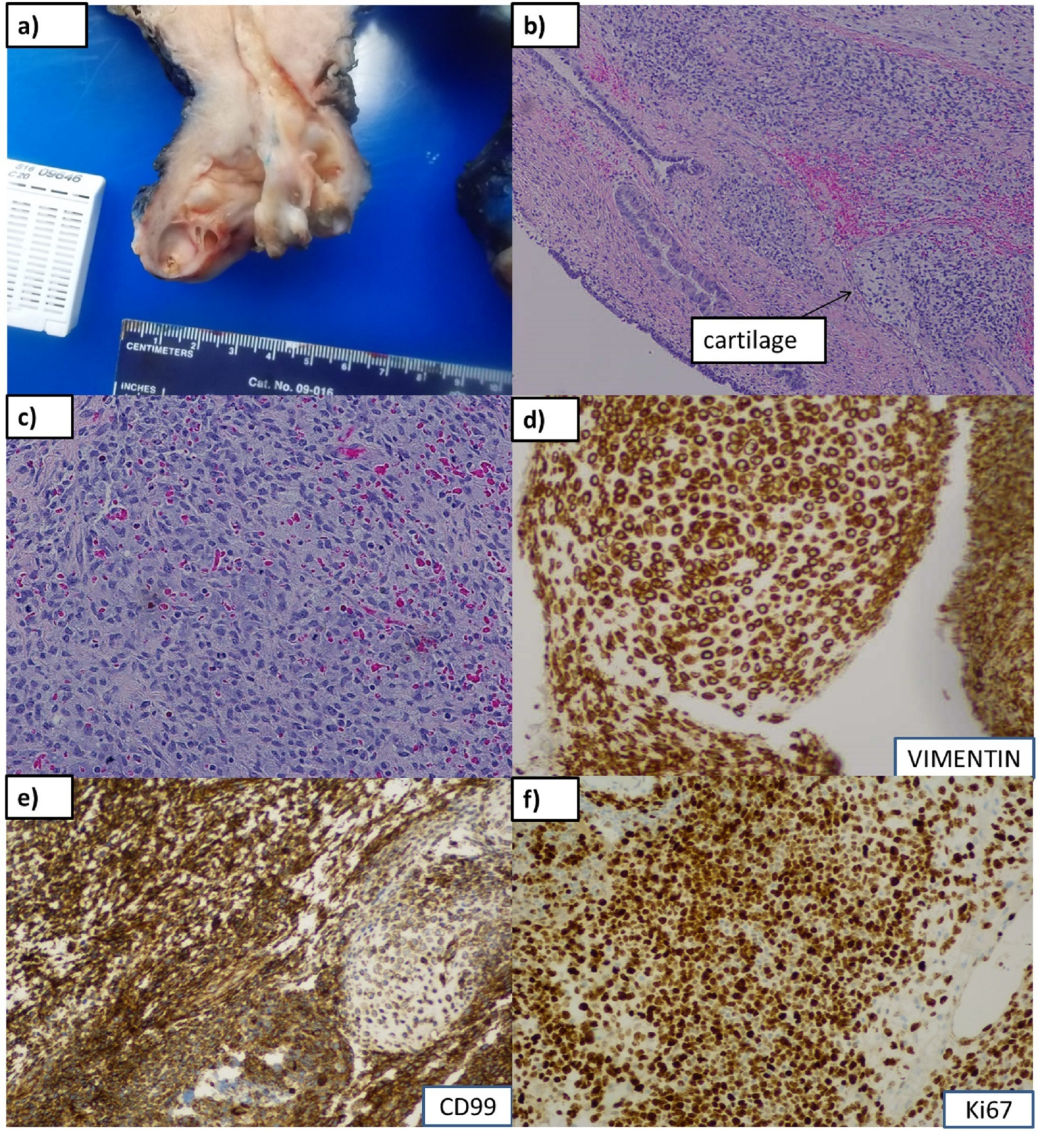
Gross and microscopic features (a): Gross polypoid mass protruding through os, 8.0 cm in greatest dimension, homogeneous, fleshy, yellow-tan to gray-pink myxoid cut surface with soft consistency. Foci of necrosis and ulceration are seen. (b), (c): Microscopic findings of primitive small cells with scant cytoplasm, islands of cartilage (arrow), alternating hypo- (with myxoid &/or edematous stroma) and hypercellular areas at 10x magnification, (b), and 20 x, (c). (d), (e), (f): Immunohistochemistry stains positive for vimentin, CD99, and Ki67, respectively.

The patient was referred to a gynecologic oncologist and further workup revealed the absence of metastatic disease. A decision for surgical intervention was made and the patient underwent a total abdominal hysterectomy, bilateral salpingo-oophorectomy, omentectomy, left pelvic lymph node biopsy, and right ureterolysis. The pathologic exam showed a tumor measuring 8 x 3 x 1 cm with a high histologic grade seen to be invading the cervical stromal connective tissue with areas of cartilaginous differentiation. No lymphovascular invasion was observed and the tumor margins were uninvolved by sarcoma. On immunohistochemistry, the cells were strongly and diffusely positive for vimentin, positive for CD99, and focally positive for myogenin and MyoD1. The findings were consistent for a diagnosis of embryonal rhabdomyosarcoma of the cervix. The pathologic stage was pT1a, i.e., tumor limited to the endocervix without myometrial invasion (Figure 1). The patient’s disease was classified as FIGO stage 1B2 (T1b, N0, M0).

Given the family history of a gynecologic malignancy at an early stage, the patient underwent genetic counseling and testing that revealed an absence of any pathogenic mutations. She was positive for two genes of unknown significance (BMPR1A and RAD51C) and no further recommendations were deemed necessary.

Based on the Intergroup Rhabdomyosarcoma Study Group (IRSG) classification [2], the patient fell into clinical group IA. Since the diagnosis is rare in the adult population, we reviewed data on treatment and outcomes in the pediatric population. Given the excellent results of multiagent chemotherapy in embryonal rhabdomyosarcoma in IRSG-conducted studies, the patient was started on adjuvant chemotherapy with the ARST0331 regimen of vincristine, dactinomycin, and cyclophosphamide (VAC) with the total duration of chemotherapy lasting 22 weeks [3]. The patient’s computed tomography (CT) scan of the abdomen and pelvis after two cycles of chemotherapy did not show any evidence of disease.

## Discussion

Rhabdomyosarcomas (RMSs) are a group of tumors that arise from immature cells destined to form striated skeletal muscle. Nearly 90% of all cases of RMSs are seen in individuals less than 25 years of age, and within this age group, 60% to 70% of the patients are less than 10 years of age. Whereas RMS accounts for 3% to 4% of all childhood cancers, it is seen with an incidence of less than 1% in adults [4]. Various sites of disease occurrence have been described, including the head and neck, lymph nodes, the genitourinary tract, extremities, trunk, and the retroperitoneum. Even though 20% of rhabdomyosarcomas in childhood arise in the genitourinary tract, the cervix is a rare site of the disease even in children and adolescents. It arises within the wall of the bladder or vagina almost exclusively in infants, whereas it can also arise elsewhere in older children. Of all cervical cancers, RMS incidence is reported to be about 1% to as low as 0.4% in some studies [5].

RMS can be subdivided into four types: embryonal, alveolar, sclerosing, and pleomorphic. The embryonal RMS is the most common type, accounting for 68% of all RMSs and is subdivided into three categories: the botryoid, spindle cell, and not-otherwise-specified (NOS). The majority of RMSs arising from the female genital tract is of the botryoid type. The term botryoid is derived from the appearance of the tumor - that of grape-like structures. Since it arises in the cervix, it grows in the narrow space and takes the appearance of a grape-like mass. On histology, the cellular pattern is variable with the presence of mitoses, pleomorphism, a cambium layer, attenuated epithelium, myxoid type stroma, and the presence of atypical rhabdomyoblasts. On immunohistochemistry (IHC), the tumor is usually seen to be positive for vimentin, desmin, actin, myoglobin, MyoD1, and myogenin. IHC is essential for a confirmatory diagnosis [6]. Focal chondroid differentiation might be apparent in some specimens, as was seen in our case [7].

Among adults, most reported cases have been seen to occur in the reproductive age group in the second and third decades of life. However, embryonal RMS has been reported in females older than 50 years of age. The most common presentation of embryonal RMS is the presence of an exophytic mass and vaginal bleeding. Of the reported cases, tumors as large as 6 cm have been seen to occur, with the smallest being about 1.5 cm. Our patient had a mass that was 8 cm, that is, larger than the mean size reported in most series [8]. Other possible presentations include amenorrhea or menorrhagia. The disease is usually noted at an early stage with the presentation of a protruding mass when it is amenable to surgical resection.

The prognosis depends on the tumor site, depth of invasion, and lymph node involvement. Tumors arising from the cervix have a better prognosis than similar tumors arising in other sites of the female genital tract. Extrapolated from findings in RMSs of the bladder and vagina, an exophytic growth pattern is associated with a more favorable prognosis than an endophytic or diffuse intramural pattern. The embryonal botryoid variant is associated with a much more favorable outcome than the alveolar and undifferentiated subtypes, which are associated with a particularly poor prognosis. An early disease stage at diagnosis is another favorable prognostic factor, whereas metastatic disease at presentation and poor response to chemotherapy are strongly associated with a poor prognosis. Adjuvant chemotherapy is decided based on a risk group of low, intermediate, or high. The regimen usually comprises of vincristine, dactinomycin, and cyclophosphamide with modifications in dosing schedule based on the prognostic group [3]. 

Since cervical sarcomas are a rare entity and histologically diverse, standardized management guidelines for the disease are lacking. Germline DICER1 pathogenic variants have been described in patients with embryonal RMS of the uterine cervix in older children and young adults, which can present as vaginal spotting or passage of myxoid, hemorrhagic solid tissue. It may be prudent to test for the mutation if suspicion of a genetic predisposition exists [9].

## Conclusions

Even though embryonal rhabdomyosarcoma is an uncommon disease of the pediatric and adolescent population, the disease must be kept in the differential diagnosis of an older female presenting with vaginal bleeding or a protruding mass. Accurate diagnosis can be established by performing appropriate immunohistochemical stains. In addition, treatment strategies for a rare disease, such as cervical RMS, are devised from experience with comparable cases having analogous tumor pathology. Reporting of the presentation and outcomes of these cases is essential in making informed therapeutic decisions for patients with RMS of the cervix. 
